# The Biochemical Composition and Antioxidant Properties of *Fucus vesiculosus* from the Arctic Region

**DOI:** 10.3390/md20030193

**Published:** 2022-03-06

**Authors:** Ekaterina D. Obluchinskaya, Olga N. Pozharitskaya, Denis V. Zakharov, Elena V. Flisyuk, Inna I. Terninko, Yulia E. Generalova, Irina E. Smekhova, Alexander N. Shikov

**Affiliations:** 1 Murmansk Marine Biological Institute of the Russian Academy of Sciences (MMBI RAS), Vladimirskaya, 17, 183010 Murmansk, Russia; obluchinskaya@mmbi.info (E.D.O.); olgapozhar@mail.ru (O.N.P.); zakharden@yandex.ru (D.V.Z.); 2 Department of Pharmaceutical Formulations, St. Petersburg State Chemical Pharmaceutical University, Prof. Popov, 14, 197376 Saint-Petersburg, Russia; elena.flisyuk@pharminnotech.com (E.V.F.); irina.smekhova@pharminnotech.com (I.E.S.); 3 Core Shared Research Facilities “Analytical Center”, St. Petersburg State Chemical Pharmaceutical University, Prof. Popov, 14, 197376 Saint-Petersburg, Russia; inna.terninko@pharminnotech.com (I.I.T.); yulia.generalova@pharminnotech.com (Y.E.G.)

**Keywords:** Arctic, *Fucus vesiculosus*, monosaccharides, phlorotannins, flavonoids, reproductive phase, seaweed, toxic metals

## Abstract

*Fucus vesiculosus* is one of the most prominent brown algae in the shallow waters of the seas of the Arctic region (Barents (BS), White (WS), Norwegian (NS), and Irminger (IS)). The aim of this study was to determine the biochemical composition of *F. vesiculosus* from the Arctic at different reproductive phases, and to evaluate the antioxidant properties of *F. vesiculosus* extracts. The amounts of monosaccharides, phlorotannins, flavonoids, and ash and the mineral composition significantly varied in the algae. A strong correlation was established between monosaccharide, phlorotannin, and flavonoid accumulation and water salinity (Pearson’s correlation coefficients *r* = −0.58, 0.83, and 0.44, respectively; *p* < 0.05). We noted a negative correlation between the antioxidant activity and the amount of the structural monosaccharides of fucoidan (*r* = −0.64). A positive correlation of phlorotannins and flavonoids with antioxidant power was confirmed for all samples. The ash accumulation was relatively lower in the sterile phase for the algae from the BS and WS. The correlation between the Metal Pollution Index (MPI) and the reproductive phases was medium with high fluctuation. Meanwhile, the MPI strongly correlated with the salinity and sampling site. The gradient of the MPI values across the sea was in the following ranking order: BS < WS < NS < IS. Taken together, and based on our data on the elemental contents of *F. vesiculosus*, we believe that this alga does not accumulate toxic doses of elements. Therefore, the Arctic *F. vesiculosus* could be safely used in food and drug development as a source of active biochemical compounds and as a source of dietary elements to cover the daily nutritional requirements of humans.

## 1. Introduction

Brown algae have been attracting more attention from researchers [1,2,3]. *Fucus vesiculosus* is one of the most prominent brown algae of the genus *Fucus*, which currently comprises 66 taxonomically accepted species. These algae prevail in shallow-water macroalgae populations, at a depth of 0.5–4 m, in sea waters with typically high salinity. Such communities form wide belts and are the habitat of epiphytic and epibenthic organisms [4]. *Fucus* species are consumed by populations of the coastal countries of Western Europe and Alaska [5]. Besides being utilized in food ingredients, *F. vesiculosus* is used in cosmetics, biofertilizers, animal feed, and in the pharmaceutical industry [6,7,8,9,10,11].

*F. vesiculosus* is a rich source of health-promoting compounds such as fucoidans, polyphenols, fucoxanthin, and essential minerals [9,12,13,14,15,16,17]. Fucoidans are polysaccharides from the Fucan family and are distinguished as bioactive compounds unique to brown algae [18]. Polysaccharides from *F. vesiculosus,* with the main monomer unit of α-1,3 or α-1,4 *L*-fucopyranosyls, contain approximately 44% fucose, 26% sulfate, and 31% ash [19,20]. Various pharmacological effects, including antioxidant [21,22], anti-obesity [23], antidiabetic [24], anti-aging, antimicrobial, antitumor, anticoagulant, and anti-inflammatory [13,25], have been reported for fucoidans. Phlorotannins are a peculiar group of polyphenolic compounds, which are found only in brown algae. These compounds have a wide range of mass and degree of polymerization [26]. The brown alga *F. vesiculosus* may accumulate up to 12% of phlorotannins (based on dry weight) [27]. Phlorotannins show numerous important biological activities such as antioxidant, antibacterial, and antidiabetic properties [9,28,29] Recently, Circuncisão et al. (2018) noted the ability of *F. vesiculosus* to accumulate relatively high amounts of minerals [30].

The amount of biologically active compounds in algae varies depending on the geographical origins [31], reproductive phase (sterile versus fertile) [32], environmental stressors, and the season of collection [33].

To the best of our knowledge, the impact of the reproductive phase and geographic location (coastal zone of the Arctic—Irminger Sea (IS), Norwegian Sea (NS), Barents Sea (BS), and White Sea (WS)) on the biochemical composition and antioxidant properties of the Arctic *Fucus vesiculosus* has not been reported yet. Therefore, the aim of this study was: (i) to determine the biochemical composition (fucose, xylose, and phlorotannin content, total content of flavonoids and ash, and the mineral composition) of *F. vesiculosus* from the Arctic regions; and (ii) to evaluate the antioxidant properties of *F. vesiculosus* extracts in vitro.

## 2. Results and Discussion

### 2.1. Fucose and Xylose Content

**Figure 1:**
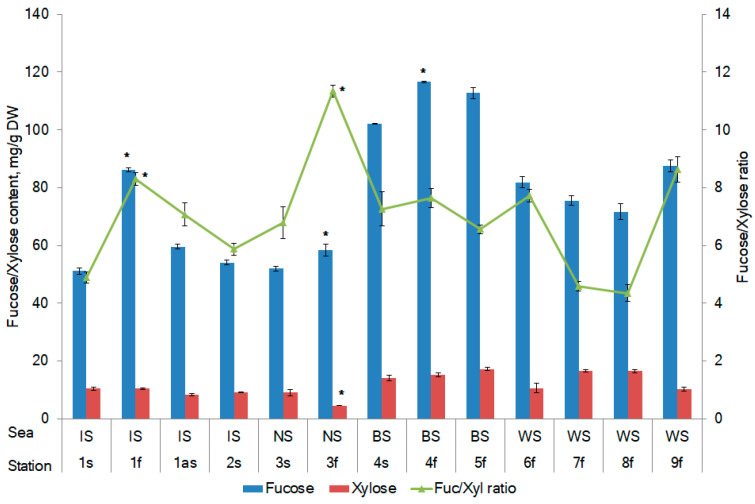
The amount of fucose and xylose in *F. vesiculosus* according to geographical location. Irminger Sea (IS), Norwegian Sea (NS), Barents Sea (BS), and White Sea (WS). s, sterile stage; f, fertile stage. Values are expressed as the mean ± SD. * *p <* 0.05 based on a comparison between fertility and sterility.

Fucose and Xylose are monomers that are always part of the fucoidans of brown algae, especially *F. vesiculosus*. The other monosaccharides of fucoidan (glucose, galactose, mannose, etc.) can be structural units in other polysaccharides and can be found in free form in algae [34,35]. Fucose is the main monosaccharide, while xylose is one of the minor monosaccharides of fucoidan from *F. vesiculosus*. The ratio of fucose to xylose can provide information about the biological activity of *F. vesiculosus* [36]. Fucose and xylose and their ratio were used in this study as an indicator of fucose-containing sulfated polysaccharide, particularly fucoidan, which is known to be abundant in the *Fucus* sp. The present study confirmed that the monosaccharide composition of fucoidans changes with the reproductive phase, but the magnitude of these changes depends on the collection area. For example, the fucose content during sporulation increased from 51.2 to 86.2 mg/g dry weight (DW) for *F. vesiculosus* from the IS, from 52.0 to 58.4 mg/g DW for the sample from the NS, and from 102.1 to 116.6 mg/g DW for the sample from the BS (Figure 1). Thus, fucose is a prevailing monosaccharide. The xylose contained in the different samples was approximately equal, and its amount was approximately 12 mg/g DW. The amount of fucose in *F. vesiculosus* was lower in the sterile phase compared with the fertile phase, whereas the xylose content did not differ significantly between the two reproductive phases (Figure 1).

**Figure 2:**
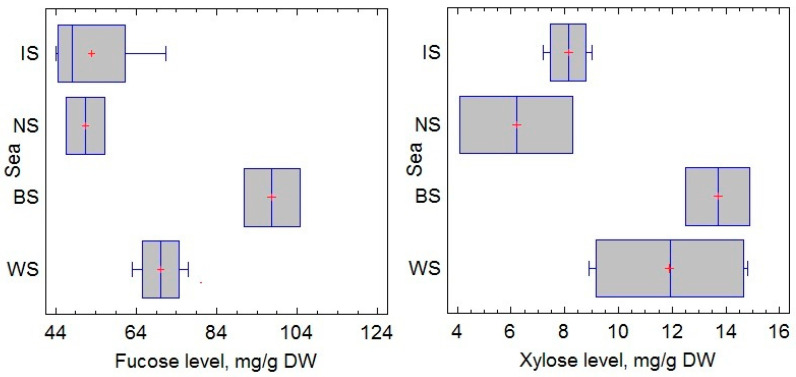
Mean levels of fucose and xylose in *F. vesiculosus* from different geographical locations. Irminger Sea (IS), Norwegian Sea (NS), Barents Sea (BS), and White Sea (WS).

The highest amounts of fucose and xylose were found in the samples from the BS (Figure 2). It was found that fucose accumulates more actively in the phase of fertility in comparison to that of sterility. The most pronounced difference (by 68%) in the amount of fucose in *F. vesiculosus,* depending on the reproductive phase, is typical for the IS. A strong negative correlation between salinity and the accumulation of fucose (Pearson’s correlation coefficients *r* = −0.58, *p* < 0.05) and xylose (*r* = −0.60, *p* < 0.05) was established. At the same time, the salinity of the sea water did not affect their ratio (*r* = 0.09, *p* < 0.05). A small positive correlation between the reproductive phase and the fucose content (Pearson’s correlation coefficients *r* = 0.23, *p* < 0.05) was detected.

The amount of biologically active compounds contained in algae is altered according to the season and related reproductive phase. According to the literature data, the maximum amount of polysaccharides accumulated in Far Eastern brown macroalgae was observed during sporulation and was accompanied by monosaccharide variation [32,37,38]. Thus, during the development of spores in *Undaria pinnatifida*, the ratio between the structural units of fucoidan changes. The proportion of galactose significantly increases and the amount of mannose decreases against the background of a constant fucose content [38]. On the contrary, galactose varies insignificantly in the polysaccharide extracted from the fertile or sterile *C. costata*, while the fraction of fucose significantly increases and mannose decreases during sporulation [37]. An increase in fucose in polysaccharides during the reproductive phase has also been noted for *Laminaria japonica* [39]. Both the sterile and fertile individuals of *F. evanescens* and *S. babingtonii* synthesize a relatively homogeneous fucoidan with a predominance of fucose, the proportion of which changes insignificantly during the development of the generative phase. The molar ratio of galactose and fucose in *C. costata* has been found to be similar in fucoidans extracted from sterile and fertile algae [32].

### 2.2. Phlorotannin Content

**Figure 3:**
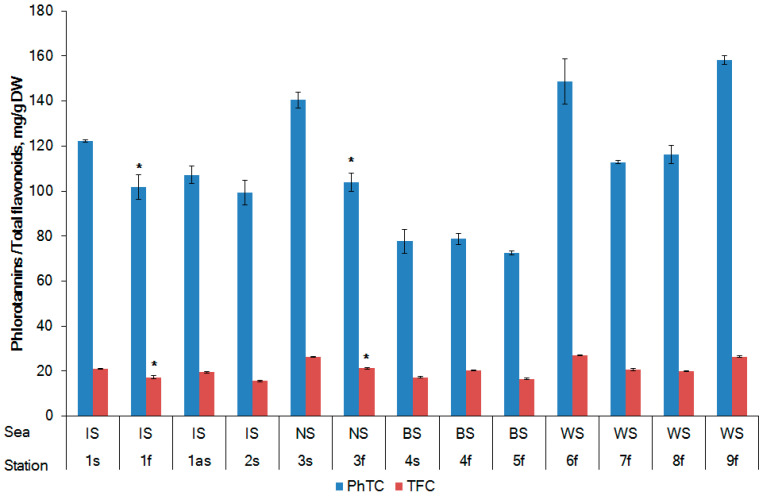
The amounts of PhTC and TFC in *F. vesiculosus* according to geographical location. Irminger Sea (IS), Norwegian Sea (NS), Barents Sea (BS), and White Sea (WS). s, sterile phase; f, fertile phase. Values are expressed as the mean ± SD. * *p <* 0.05 based on a comparison between fertility and sterility.

Significant variations were found in the phlorotannins content (PhTC) of the samples of *F. vesiculosus* from different geographic locations, ranging from 72.4 to 158.1 mg phloroglucinol equivalent (PhE) per gram of DW algae (Figure 3). *F. vesiculosus* from the BS showed a lower value of 77.7 mg/g DW for the samples in the sterile phase when compared with the values of 122.3 and 140.5 mg/g DW for the samples in the same reproductive phase from the IS and NS, respectively. A similar trend was observed for *F. vesiculosus* in the fertile phase. The PhTC in the algae from the BS was lower (78.8 mg/g DW) when compared to the PhTC values of 101.9 and 103.9 mg/g DW in the algae from the IS and NS, respectively. A statistically significant difference in PhTC for the samples collected in the WS (St. 6 and 9 vs. St. 7 and 8) was noted (*p* < 0.01).

**Figure 4:**
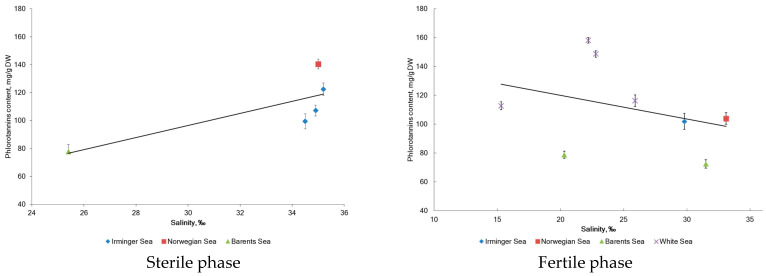
Influence of the reproductive phase and salinity on the accumulation of phlorotannins in *F. vesiculosus.*

After analyzing the reproductive phases of *F. vesiculosus* from three different localities (St. 1 from the IS, St. 3 from the NS, and St. 4 from the BS), it was found that the accumulation of phenolic compounds depended on the sea water salinity of the sampling stations (Figure 4). A strong positive correlation between salinity and the accumulation of phlorotannins (Pearson’s correlation coefficients *r* = 0.83, *p* < 0.05) and flavonoids (*r* = 0.44, *p* < 0.05) was observed. Our data support the previous results of Pedersen (1984), who reported an increase in the phenolic content in *F. vesiculosus* according to the salinity of the algal habitats [40]. At the same time, the water temperature also affected the phlorotannin and flavonoid content (*r* = −0.22 and −0.32, respectively; *p* < 0.05).

Brown algae generally contain higher amounts of polyphenols than red and green algae [41]. Brown algae are a valuable source of polyphenols, among which phlorotannins represent their principal phenolic constituents in Fucaceae [42]. Fucaceae polyphenols are very susceptible to inter-species variations as well as differences in collection site, season, water salinity, water depth, etc. [43,44]. The highest amount of phenolics (approximately 58 mg/g DW) was observed in *A. nodosum* and *F. vesiculosus* growing in the mid-tide zone, while a lower phenolic content (43 mg/g DW) was observed in *F. serratus*, growing in the lower intertidal level. Other species such as *F. spiralis* and *P. canaliculata,* growing in the upper level of the intertidal zone, had the lowest phenolic content (39 and 34 mg/g DW) [45]. According to Ragan and Jensen (1978), the polyphenol content of *F. vesiculosus* collected in Trondheimsfjord (Norwegian Sea) was minimal (80–100 mg/g DW) at the end of spring, during the period of fertility, and was at a maximum (110–130 mg/g DW) during the winter [46]. These data are in agreement with ours for the samples collected in the Norwegian and Irminger Seas (Figure 3). This phenomenon could be associated with the protective role of polyphenols during the winter season. Later, Connan et al. (2004) investigated phlorotannins in Fucaceae spp. (including *F. vesiculosus*) collected from the northern coast of Brittany (France, North Sea). For these southern species, the phlorotannin peak was observed during the summer, matching the higher solar exposure period. The production of phlorotannins by seaweeds is positively correlated with UV radiation [43,47]. This fact supports the UV-protective functions invoked for phlorotannins [48].

Flavonoids represent another group of active compounds in Fucaceae spp. [27]. Different biological activities are attributed to algal flavonoids, including antioxidant properties, scavenging of reactive oxygen species, and inhibition of lipid peroxidation [49]. The total flavonoid content (TFC) in the *F. vesiculosus* samples was quantified as quercetin equivalents (QE), and the results are illustrated in Figure 3. Depending on the geographical regions and reproductive phase, the TFC in *F. vesiculosus* varied from 15.6 to 26.4 mg QE/g DW. Our results are similar to the data of Cox et al. (2010), who reported the TFC in six Irish edible seaweeds being in the diapason of 7.6–42.5 mg QE/g DW. The samples of *F. vesiculosus* from the IS and NS had a higher TFC in the sterile phase compared to that in the fertile phase (Figure 3). Previously, a higher TFC was found in *Saccharina latissima*, cultivated in the NS (inner Danish waters) during the period of November–January (sterile phase) [50]. The TFC in algae collected in the BS was less susceptible to variations, depending on the reproductive phase (17.2 and 20.3 mg QE/g DW for the fertile and sterile phases, respectively). Differences in the PhTC levels between the fertile and sterile algae from the BS were statistically insignificant (Figure 3).

### 2.3. Antioxidant Activity

The antioxidant activity for the alga extracts is expressed as the antiradical power (ARP). The ARP is the reciprocal of the IC_50_, which defines the concentration of the extract required to scavenge 50% of the 2,2-diphenyl-1-picrylhydrazyl (DPPH) radicals.

**Figure 5:**
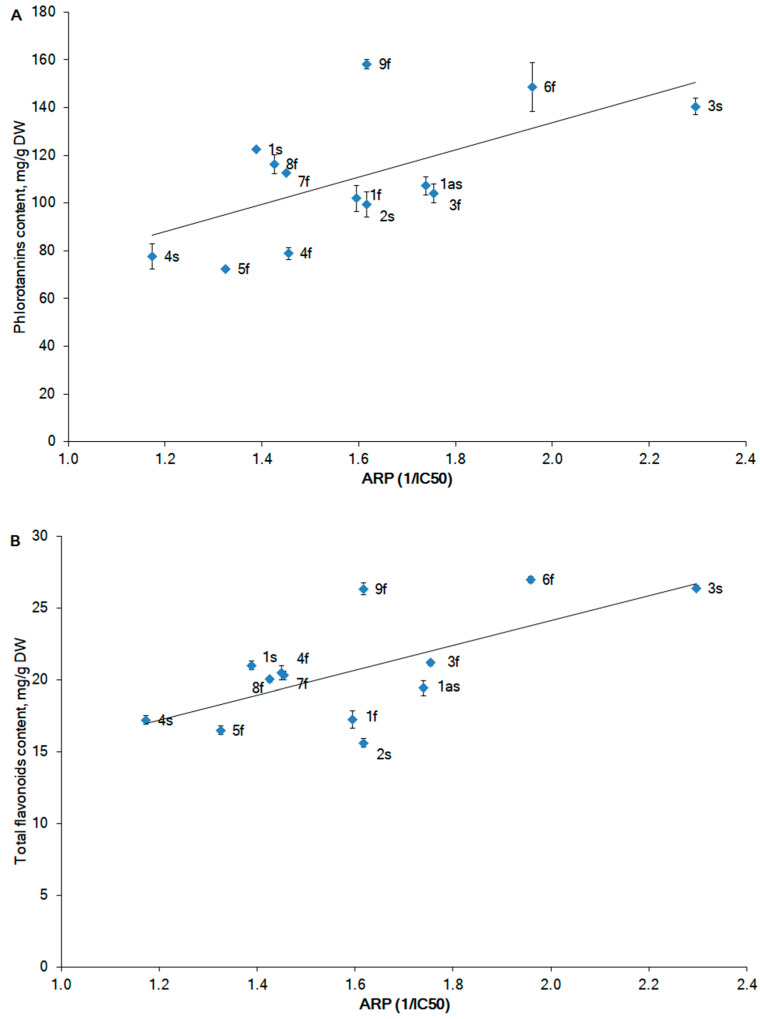
The correlation between the ARP and the phlorotannin content (**A**) and total flavonoid content (**B**) in *Fucus vesiculosus* according to geographical location. The sample number corresponds to the sampling station (see Figure 3).

The ARPs ranged from 1.2 to 2.3 (Figure 5), with the highest ARP value being found in the alga sample (St. 3s) from the NS collected in the sterile phase, and the lowest value in the sample (St. 4s) from the BS, also collected in the sterile phase (Figure 5A). An increase in the ARP in the fertile phase by 15–24% compared to the sterile phase was observed for algae from the IS and BS, while for the samples from the NS, the situation was the opposite. The increase in the ARP was 34% more prominent in the sterile phase (3s) compared to the fertile phase (3f). A strong positive correlation of ARP and PhTC (Pearson’s correlation coefficients *r* = 0.64, *p* < 0.05) (Figure 5A) was noted. Our results are consistent with previous studies that reported a direct correlation between the DPPH scavenging activity and the polyphenolic compounds of algal extracts [50,51,52]. Flavonoids positively contribute to the ARP. A correlation between the ARP and TFC of a similar fashion (*r* = 0.66, *p* < 0.05) was noted (Figure 5B).

The structural units of fucoidan, fucose, and xylose negatively correlated with the ARP (Pearson’s correlation coefficients *r =* −0.42 and −0.58, respectively), which suggests a negative impact of the fucoidan content on the radical scavenging activity of extracts of *F. vesiculosus*. However, fucoidan has been reported to have radical scavenging activity in several studies [24,53,54]. In previous studies, fucoidan was extracted from different algae and was not purified. It is likely that other compounds in crude fucoidan such as specific phenolic compounds, ascorbic acid, and proteins may contribute to the radical scavenging activity. We believe that future studies with highly purified fucoidan are required to clarify the impact of this polysaccharide on radical scavenging activity.

### 2.4. Ash Contennt

According to previous studies [30,55,56,57], the ash content of macroalgae varies significantly, depending on the species, geographic location, and reproductive phase. In our study, the ash content (DW) in the *F. vesiculosus* samples (Table 1) showed average values of 23.3% and 21.9% for the IS in the sterile and fertile phases; 20.2% and 18.5% at Cape Sudspissen for the NS in the sterile and fertile phases; 21.2%. and 28.5% for the BS in the sterile and fertile phases, respectively; and 19.1–20.3% for the WS in the fertile phase, which is within the previously reported range for algae and algal food (8–44% DW) [58,59,60]. The ash content in the *F. vesiculosus* samples collected from the IS and NS was higher in the sterile than in the fertile phase, while the ash in the samples from the BS was higher in the fertile than in the sterile phase. In a previous study, the ash content of *F. vesiculosus* from the Baltic Sea ranged from 14.2% to 21.4% and did not have a statistically significant correlation with the reproductive phase [61]. Other seasonal studies of Phaeophyceae algae [62] have not revealed a general trend in the ash content, since in some species, the percentage of ash is significantly higher in the sterile phase, while in others, it is higher in the fertile phase. Interesting results were reported by Paiva et al. (2018) for *F. spiralis* collected around the Azorean Islands. The maximum ash value was found in the sterile phase (29.6% DW) and the minimum (22.4% DW) in the fertile phase for algae around Santa Maria Island, while for the São Miguel Island samples, a maximum ash value was found in the fertile phase (25.4% DW) and a minimum (22.7% DW) in the sterile phase [55]. Although the high variability in the ash content of algae could be explained, at least in part, by spatial and temporal fluctuations in the mineral content of seawater in the Arctic and North Atlantic regions, the impact of the reproductive phase on the accumulation of ash requires future investigations.

### 2.5. Elemental Concentrations

**Figure 6:**
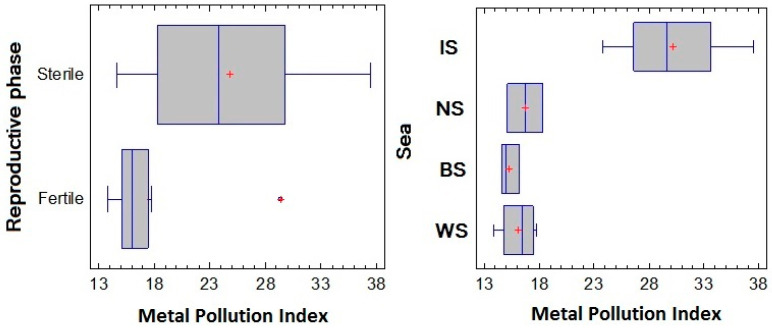
The accumulation of toxic metals calculated as MPI vs. the reproductive phase and the sampling sites of *F. vesiculosus*. Irminger Sea (IS), Norwegian Sea (NS), Barents Sea (BS), and White Sea (WS).

The measured elemental concentrations (mg/kg DW), the range (minimum and maximum concentration) for the elements, and the LOQ of the method are summarized in Table 1 for each alga sample. The elemental concentrations varied according to the seaweed sampling stations and reproductive phase. The Al, Fe, Ca, Cu, Mg, and Zn levels in *F. vesiculosus* from the IS (St. 1 and St. 2) were significantly higher than those found in the alga samples collected in the other regions. The highest content of Co was found in the samples from the NS and WS. *F. vesiculosus* from the BS contained the highest concentrations of total As. The Sr concentration in *F. vesiculosus* from the BS and WS was slightly lower than that in the samples from the IS and NS. High Sr levels were also found in *F. vesiculosus* from the IS and WS (St. 1a and St. 9). The levels of Ba and Rb were not markedly different between the sampling stations. In our study, we did not detect Pb, Cd, Cr, or Ni in *F. vesiculosus* collected at different stations. These elements were under the limit of quantification (LOQ), which corresponds to a natural background. Similar results for the Pb content in the *F. vesiculosus* samples from the Barents and White Seas were obtained previously [63]. The data on the accumulation of toxic metals calculated as the Metal Pollution Index (MPI) versus the sampling stations and the reproductive phase are shown in Figure 6.

The MPI values ranged from 13.8 to 37.5, showing differences in content across the sampling sites and reproductive phases. The lowest mean MPI (15.3) was found in the samples from the BS, while the highest mean MPI (29.7) as well as the greatest dispersion of data was found in the samples from the IS, indicating the most significant fluctuations of metal concentration in the samples (Figure 6). A gradient of MPI values across the sea was established, ranked in the following order: BS < WS < NS < IS. A strong Pearson’s correlation was found for the MPI value versus the sampling site (*r* = 0.79, *p* <0.05), while a medium Pearson’s correlation was found for the MPI value versus the reproductive phase (*r* = 0.42, *p* < 0.05).

The Pearson’s correlation coefficients (*r*), calculated for individual metals, are presented in Table 2. A strong positive correlation (*r* > 0.9) was identified between Cu and Fe and between Cu and Zn. Positive correlations (0.9 > *r* > 0.5) were noted for Al versus Cu, Fe, Mg, Sr, and Zn; Cu versus Mg; Fe versus Mg and Zn; Ba versus Mn; Co versus Cu, Sr, and Zn; and Mg versus Mn. Similarly, negative correlations (−0.8 > *r* > −0.5) occurred only for As versus Ba, Mg, Mn, and Rb.

Algae consumption enriches the daily diet with proteins, fatty acids, vitamins, and minerals and has become increasingly popular in Western countries [64]. The increased metal pollution in the marine environment has put a significant burden on the ecosystem and has become a risk factor for the accumulation of toxic elements in algae. Some countries have implemented limits for heavy metals in algae dietary food. Thus, in France, a special list of algae for human consumption specifies the upper limits for the amounts of Pb, Cd, Sr, Hg, As, and I [65]. The amount of some metals such as Pb, As, Cd, and Hg in food algae is limited in Russia [66]. Provisional tolerable weekly and monthly intakes (PTWI and PTWM) for several elements are recommended by the Joint FAO/WHO Expert Committee on Food Additives [67,68,69]. The upper intake level (UL) of the elements approved by the European Food Safety Authority (EFSA) is calculated for the average adult body weight (BW) of 70 kg [70]. In particular, the FAO/WHO Joint Expert Committee on Food Additives has defined the PTWI for Al from all sources as 70 mg [67]. The WHO indicates a PTWI of 1.05 mg for inorganic As [68]. EFSA has established a UL of 2500, 25, and 5 for Ca, Zn, and Cu, respectively [70]. The Federal Center for Hygiene and Epidemiology of Rospotrebnadzor of Russia also has a UL for certain elements [71].

To understand the benefits or risks of the consumption of *F. vesiculosus* collected from different stations, we calculated the amount of certain elements in a daily dietary dose of algae. A dose of 3.3 g (DW) of algae was considered as the average daily consumption, and the maximum amount of algae in a single serving was 12.5 g (DW) [72]. In Table 3, we summarize the data on the sampling stations and reproductive phases at which the maximal concentration of a particular element was detected in the algae. Then, we calculated the maximal amount of elements consumed with 3.3 g and 12.5 g of algae and subsequently compared this with the risk estimations for a 70 kg man [67,68,69,70] and with the nutritional requirements [70,71].

The comparison between intake and relevant UL reported by EFSA [70] shows that a 3.3–12.5 g daily consumption of *F. vesiculosus* in the sterile phase from the IS (St. 1), with the highest Ca level (30 g/kg DW), corresponds to a daily intake of 0.099–0.38 g of this metal. This intake alone is equal to approximately 4–15% of the tolerable daily dose (2.5 g) for Ca (Table 3). The daily consumption of 3.3–12.5 g of *F. vesiculosus* with the highest Cu (16.6 mg/kg DW) in the sterile phase from the IS (St. 2) corresponds to a daily intake of 0.05–0.21 mg of this metal. With this dose of algae, approximately 1.1–4.1% of the tolerated daily intake (5 mg) of Cu is consumed. In this case, the daily consumption of 12.5 g of *F. vesiculosus* in the sterile phase from the IS (St. 2) with the highest Zn concentration (107 mg/kg DW) will lead to a daily intake of 1.33 mg of this element. This corresponds to approximately 5.3% of the tolerable daily dose (25 mg) for Zn. The daily consumption of 3.3–12.5 g of *F. vesiculosus* in the fertile phase from the IS (St. 1), with the highest Al level (723 mg/kg DW), corresponds to a daily intake of 2.4–9.0 mg of this metal. This amount of Al represents approximately 24%–90% of the tolerable daily dose (10 mg) [67]. A daily dose of 3.3–12.5 g of *F. vesiculosus* from the BS (St. 4) in the sterile phase contains 0.19–0.72 mg of total As. Such doses of algae correspond to 127%–478% of the tolerable daily dose for inorganic As.

Worthy of note is the fact that in this study, As was measured as total As. High As levels have been reported in the literature for several algae [73,74,75]. It is necessary to note that As is found in marine biota mainly in the form of organic compounds (in particular, As sugars). Although inorganic forms of tri- (AsIII) and pentavalent (AsV) are toxic, their organic derivatives (arsenobentaine (AsB), arsenosugar (As sugar), arsenocholine (AsC), arsenolipids, methylarsinate (MMA), and dimethylarsinate (DMA)) are of low toxicity. The toxicity of arsenic compounds, according to the LD_50_, decreases from inorganic to organic: AsIII (14) > AsV (20) > MMA (700–1800) > DMA (700–2600) > AsC (>6500) > AsB > As sugar (>10,000) [76]. While the toxicity of arsenolipids has not been established, arsenobetaine and other organic arsenic compounds belong to group 3 (substances not classified for carcinogenicity) according to the classification of the International Agency for Research on Cancer [77].

Thus, summarizing our data on the elemental content of *F. vesiculosus* collected from different regions, we can suggest this alga as non-toxic and as a source of dietary elements that cover daily nutritional requirements.

## 3. Materials and Methods

### 3.1. Alga Sample Collection

Alga samples were collected in the coastal zone of the Artic, including the Irminger Sea, Norwegian Sea, Barents Sea, and White Sea (Figure 1), according to standard protocols [33,78]. Alga samples were taken at low tide, at a 0.6–1.0 m depth, in the fertile and sterile phases in 2019. The number, size, or fresh mass of the receptacles of the alga samples was used as a proxy for *Fucus* fertility [79,80]. The reproductive phases of the algae were determined by the presence of receptacles (reproductive organs): all alga samples collected in the summer period retained mature receptacles. The autumn samples did not contain receptacles. The temperature and salinity were measured with a thermometer TL-4 (Thermopribor JSC, Klin, Russia) and a refractometer RHS-10ATC (Kelilong Electron Co., Ltd., Fuan, China), respectively. Freshly collected algae were washed thoroughly in seawater and transported to the laboratory immediately. The alga samples were identified by Dr. E. Obluchinskaya, and the voucher specimens were deposited in the Collection of the Zoobentos Laboratory (MMBI RAS). The alga samples were dried at 20 °C, ground up to a 1 mm particle size using a non-metallic mill (CT 293 Cyclotec, Foss, Hilleroed, Denmark), and then kept at room temperature until analyses.

### 3.2. Sampling Stations

**Figure 7:**
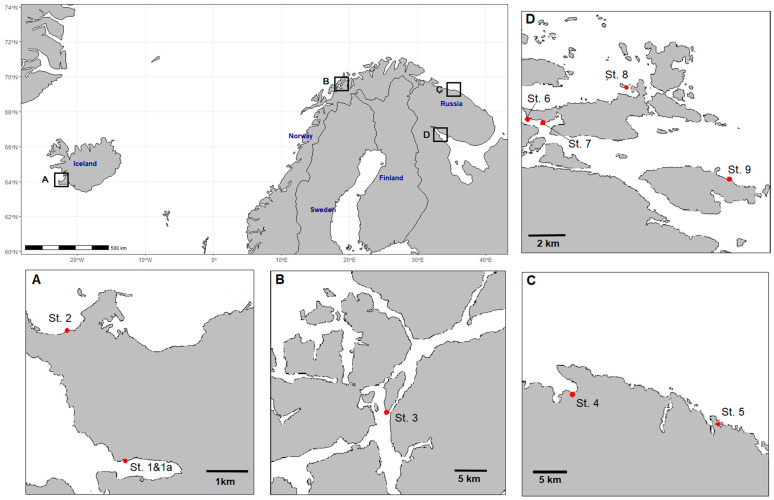
The locations of the alga sampling stations: A, Irminger Sea; B, Norwegian Sea; C, Barents Sea; D, White Sea.

The sampling areas and stations are shown in Figure 7. Samples of *F. vesiculosus* from different geographical locations around Iceland, Norway, and Russia were collected for the study: The Irminger Sea, Fossvogur Bay, and Seltjarnarnes Peninsula (Stations 1 and 2; Figure 7A); the Norwegian Sea, Cape Sudspissen (St. 3; Figure 7B); the Barents Sea, Teriberskaya and Zelenetskaya bays (St. 4 and 5, Figure 7C); the White Sea, Kandalaksha Bay Islands (St. 6–9; Figure 7D and Table 4). In the sampling areas, there were no industrial enterprises or visible pollution with garbage or oil products. An anthropogenic load at the stations of the Barents Sea and the White Sea was practically absent (St. 4–9). Stations St. 1, 1a, 2, and 3 are located near cities.

### 3.3. Chemicals

Nitric acid solutions were prepared by the dilution of 65% stock solution (PanReac, Darmstadt, Germany). Multi-element standard solutions were prepared by the dilution of 1000 µg mL^−1^ of Multi-Element Calibration Standard 3 (concentration 10 µg/mL) (PerkinElmer, Waltham, MA, USA) in the range of 0.1–1.0 mg/L. All glassware pieces were previously decontaminated with 10% v/v HNO_3_ for 24 h.

The Folin–Ciocalteu reagent, quercetin, phloroglucinol, fucose, xylose, and 2,2-diphenyl-1-picrylhydrazyl (DPPH) were from Sigma-Aldrich (St. Louis, MO, USA). The other analytical-grade chemicals and solvents used for the extraction and assay were purchased from local chemical suppliers. All reagent solutions were prepared using ultrapure water (resistivity of 18.2 MΩ cm) obtained from a Milli-Q purification system (Millipore, Bedford, MA, USA).

### 3.4. Fucose–Xylose Composition

The amounts of fucose and xylose were determined after hydrolysis of the dried seaweed samples [16]. The hydrolysate was cooled in an ice water bath and centrifuged at 2300× *g* (3500 rpm) for 15 min at 25 °C using a medical laboratory centrifuge (CM-6M, Elmi Ltd., Riga, Latvia). The supernatant was neutralized to pH 7 with 2 M NaOH. The resulting samples were analyzed by HPLC using an LC-10A chromatograph with an RID-10A detector (Shimadzu Corp., Kyoto, Japan) according to the method in [81]. Samples were separated on a Shodex Asahipak NH2P-50 4E 250 × 4.6 mm column (Showa Denko Co., Tokyo, Japan) at 50 °C. A mixture of 0.25 M orthophosphoric acid and acetonitrile (20:80) was used as the mobile phase, at a flow rate of 1.0 mL/min. Fucose and xylose were used as the reference compounds.

### 3.5. Determination of the Phlorotannin Content, Total Flavonoids, and Antioxidant Activity

The samples of *F. vesiculosus* were extracted following the method of [82], with some modifications. The powdered sample (2 g) was macerated three times with 50 mL of aqueous methanol (60% *v/v*), in a dark place and at room temperature, for 24 h under continuous stirring at 200 rpm using the Multi Bio RS-24 (Biosan, Riga, Latvia). The mixture was centrifuged (3500 rpm, 10 min), filtered, and combined. The filtrate was concentrated to dryness under vacuum on a rotary evaporator IR-1m (PJSC Khimlaborpribor, Klin, Russia), and the residue was dissolved in 25 mL volumetric flasks with 60% *v/v* aqueous methanol. The phlorotannin content (PhTC), the total flavonoid content (TFC), and the DPPH scavenging activity were analyzed in triplicate.

The amount of PhTC was determined with the Folin–Ciocalteau reagent according to [83]. Briefly, 0.5 mL of the solution of *Fucus* extracts was mixed with 2 mL of the Na_2_CO_3_ solution (200 mg/mL) and 10 mL of ultrapure water; after 5 min, 0.5 mL of the Folin–Ciocalteau reagent was added. The solutions were mixed and incubated at room temperature in dark conditions for 2 h. The precipitate formed was removed by centrifugation (PE-6900, Ekros-Analytica LLC, St. Petersburg, Russia) at 3500 rpm for 10 min. Finally, the absorbance was measured at 750 nm (Shimadzu UV 1800, Shimadzu, Kyoto, Japan) and compared to a phloroglucinol calibration curve spectrophotometer. The results are expressed as milligrams of phloroglucinol equivalent per gram (PhE/g) of *F. vesiculosus* DW. The TFC was measured by a colorimetric assay [84], with some modifications. Briefly, a 0.5 mL aliquot of the alga extract was added to a volumetric flask containing 2 mL of water and 0.15 mL of an aqueous NaNO_2_ solution (5 g/100 mL). After 5 min, 0.15 mL of an aqueous AlCl_3_ solution (10 g/100 mL) was added. After a further 6 min, 1 mL of M NaOH was added, and the mixture in the reaction flask was diluted to volume with the addition of 1.2 mL of ddH_2_O and then thoroughly mixed. The absorbance of the mixture was measured at 415 nm after 30 min against a blank using a UV-Vis spectrophotometer Shimadzu UV 1800 (Shimadzu, Kyoto, Japan). The TFC was expressed as milligrams of quercetin equivalent per gram (QE/g) of *F. vesiculosus* DW. A blank was prepared, as described above, except that aluminum chloride was replaced with aqueous methanol.

The DPPH scavenging activity was analyzed according to Brand-Williams et al. (1995) [85], with some modifications [51]. Briefly, 1 mL of the extract or standard was mixed well with 1.5 mL of H_2_O and 0.5 mL of a 100 µM DPPH methanolic solution in a test tube. L-ascorbic acid was used as the reference standard. The same concentration of methanol and DPPH was used as the control without extract solution. The reactive solutions were left in the dark at room temperature for 30 min. Then, the absorbance at 517 nm was taken using a Shimadzu UV 1800 UV–Vis spectrophotometer (Shimadzu, Kyoto, Japan). The percent of remaining DPPH ($\%DPPH_{R}^{\cdot }$) of different samples was calculated as follows:

(1) $$\%DPPH_{R}^{\cdot }=\frac{DPPH_{T}^{\cdot }}{DPPH_{0}^{\cdot }}\times 100$$

where $DPPH_{0}^{\cdot }$ is the concentration of DPPH at time zero (initial concentration), and $DPPH_{T}^{\cdot }$ is the concentration of DPPH after 30 min.

The percentage of remaining DPPH was plotted against the sample/standard concentration to obtain the IC_50_ value, which represents the concentration of the extract or standard antioxidant (mg/mL) required to scavenge 50% of the DPPH radical in the reaction mixture. Its reciprocal, the antiradical power (ARP, ARP = 1/IC_50_), was also calculated for each of the sample extracts [51].

### 3.6. Ash Content

Samples (0.5–1.5 mm) were dried at 105 °C for 24 h for complete drying, and then desiccated during cooling. After cooling, 1 g of the dried samples was placed in a crucible and transferred to a muffle furnace for 24 h at 575 °C to ensure complete combustion. The sample crucibles were cooled in a desiccator and weighed after cooling.

### 3.7. Elemental Analysis

A PerkinElmer^®^ Optima™ 8000 Model inductively coupled plasma optical emission spectrophotometer (ICP-OES) (PerkinElmer, Inc., Shelton, CT, USA) was used to quantify the metal ions in the alga tissue samples [86]. The instrument was optimized daily before the measurements and operated as recommended by the manufacturer. The instrumental parameters were: Plasma gas flow, 10 L/min; auxiliary argon flow rate, 0.2 L/min; nebulizer gas flow rate, 0.7 L/min; plasma power, 1300 W; sample flow rate, 1.5 mL/min. The microwave digestion unit Speedwave Entry Two (Berghof, Eningen unter Achalm, Germany) was used for the decomposition of the plant samples before analyses by ICP-OES [87]. All measurements were performed using argon gas to form plasma. The wavelengths (nm) were: Al 396.153; As 188.979; Ba 455.403; Bi 223.061; Ca 317.933; Cd 214.440; Co 238.892; Cr 267.716; Cu 327.393; Fe 238.204; Mg 279.077; Mn 257.610; Ni 231.604; Pb 220.353; Rb 780.023; Sr 407.771; Zn 213.857.

Sample aliquots of approximately 400 mg were digested using 5 mL of HNO_3_. Blank solutions were prepared by applying the same procedure and reagent solutions without sample. The digestion program consisted of three steps: Room temperature to 150 °C in 5 min; 150–190 °C in 10 min; 190–75 °C in 15 min. After cooling to room temperature, the digested material was transferred to a 50 mL volumetric flask, and the volume was set with ultrapure water. Analytical signals were measured as emission intensity values.

### 3.8. Metal Pollution Index

The Metal Pollution Index (MPI) [78,88] is a mathematical model that summarizes the values for all toxic metals, calculated as the mean of values for the metals considered and expressed as follows:

*MPI* = (*M*_1_ × *M*_2_ × … × *M_n_*)^1*/n*^ (2)

where *M_n_* is the concentration of the metal, *n,* in the sample in milligrams per kilogram of DW.

The nutrimental importance of essential elements was assessed on the basis of nutritional requirements (European Food Safety Authority) [70]. The health risk due to the toxic elements present in seaweeds was estimated using risk estimators [67,68,69,70,71].

### 3.9. Statistical Analysis

All statistical analyses were performed with STATGRAPHICS Centurion XV (StatPoint Technologies Inc., Warrenton, VA, USA). The data are expressed as the mean ± standard deviation (±SD), and the error bars in the figures indicate the standard deviation. The differences between the means were analyzed by ANOVA, followed by Tukey’s post hoc test. A significant difference was considered at a level of *p* < 0.05. Pearson’s correlation coefficients were used to establish the relationship between the content of representative compounds and antioxidant capacity. Multiple regression and multivariate data analysis such as the partial least squares coefficient method were carried out.

## 4. Conclusions

To the best of our knowledge, this is the first study in which the impact of the reproductive phase and geographic location (coastal zone of the Arctic—Irminger Sea (IS), Norwegian Sea (NS), Barents Sea (BS), and White Sea (WS)) on the biochemical composition and antioxidant properties of the Arctic *Fucus vesiculosus* was evaluated. The biochemical composition of *F. vesiculosus* significantly varied. The highest amounts of fucose and xylose were found in the algae from the BS collected in the fertile phase. A strong correlation was established for monosaccharide, phlorotannin, and flavonoid accumulation and water salinity (Pearson’s correlation coefficients *r* = −0.58, 0.83, and 0.44, respectively; *p* < 0.05). We noted a negative correlation between radical scavenging activity and the amount of structural monosaccharides of fucoidan. However, future studies with purified fucoidan are necessary to clarify this observation. The positive correlation of phlorotannins and flavonoids with ARP was confirmed for all samples. The ash accumulation was relatively lower in the sterile phase for the algae from the BS and WS. The correlation between the MPI and reproductive phases was medium with a high fluctuation. Meanwhile, the MPI was strongly correlated with the salinity and sampling site. The gradient of MPI values across the seas was ranked in the following order: BS < WS < NS < IS. We noted a correlation between the accumulation of several individual metals as well. Taken together, based on our data on the elemental content of *F. vesiculosus* collected from different seas of the Arctic region, we believe that this alga does not accumulate toxic doses of elements. Thus, *F. vesiculosus* could be used safely in food and drug development, as a source of active biochemical compounds and as a source of dietary elements, to cover the daily nutritional requirements of humans.

## Figures and Tables

**Table 1:** The concentrations of elements (mg/kg DW) in the samples of *Fucus vesiculosus* (mean ± SD, *n* = 3).

Element	LOQ	Range	St. 1		St. 1a	St. 2	St. 3		St. 4		St. 5	St. 6	St. 7	St. 8	St. 9
			s/f	∆	s	s	s/f	∆	s/f	∆	f	f	f	f	f
Al	1.6	28–724	423 ± 40	*	183 ± 36	688 ± 124	54 ± 8	*	45 ± 1	*	54 ± 12	95 ± 6	88 ± 5	42 ± 9	57 ± 3
			724 ± 27	↑			28 ± 3	↓	62 ± 3	↑					
As	6.3	23–58	35 ± 2		30 ± 4	28 ± 2	39 ± 1	*	58 ± 2	*	40 ± 1	30 ± 3	31 ± 2	36 ± 1	22 ± 1
			32 ± 1	↓			48 ± 2	↑	44 ± 1	↓					
Ba	0.016	10–24	13 ± 0.1		13.8 ± 0.8	12 ± 0.2	10.2 ± 0.1		11.0 ± 0.2		10.6 ± 0.2	18 ± 1	20 ± 0.3	16 ± 0.2	24 ± 2
			10 ± 0.3	↓			9.7 ± 0.1	↓	10.2 ± 0.1	↓					
Ca	1.9	9756–30,093	30,093 ± 100	*	15,228 ± 230	18,278 ± 210	20,832 ± 315	*	11,430 ± 188	*	9890 ± 20	11,922 ± 205	11,666 ± 290	20,065 ± 260	11,592 ± 470
			18,436 ± 265	↓			9756 ± 113	↓	12,015 ± 389	↑					
Co	0.12	0.4–2.0	1.73 ± 0.03		1.56 ± 0.09	1.95 ± 0.38	1.38 ± 0.02		1.09 ± 0.01	*	0.59 ± 0.05	0.61 ± 0.03	0.92 ± 0.10	0.39 ± 0.03	2.01 ± 0.20
			1.49 ± 0.10	↓			1.46 ± 0.95	↑	0.60 ± 0.02	↓					
Cu	0.37	0–16.6	3.63 ± 0.06		2.79 ± 0.15	16.60 ± 0.25	1.22 ± 0.01	*	<LOQ	*	1.67 ± 0.02	0	0	0	1.11 ± 0.2
			4.44 ± 0.30	↑			0.42 ± 0.10	↓	0.91 ± 0.10	↑					
Fe	0.098	52–2217	946 ± 10		258 ± 17	2217 ± 123	93 ± 4	*	86 ± 1	*	273 ± 18	325 ± 29	385 ± 30	216 ± 70	202 ± 6
			1007 ± 43	↑			52 ± 4	↓	128 ± 2	↑					
Mg	1.7	7518–11,571	9793 ± 40	*	8985 ± 120	11,474 ± 338	8277 ± 34	*	7634 ± 61	*	8148 ± 112	10,152 ± 25	9535 ± 80	8442 ± 55	9871 ± 58
			11,571 ± 166	↑			7518 ± 9	↓	9821 ± 258	↑					
Mn	0.058	58–176	125 ± 2	*	118 ± 8	88 ± 3	84 ± 1	*	81 ± 1	*	68 ± 2	91 ± 3	156 ± 4	58 ± 3	142 ± 9
			142 ± 3	↑			70 ± 3	↓	75 ± 2	↓					
Rb	0.55	5–29	24 ± 0.1	*	22 ± 1.0	20 ± 0.3	19 ± 0.2	*	4.7 ± 0.4	*	20 ± 0.4	25 ± 1.0	22 ± 0.6	29 ± 0.3	29 ± 1.0
			29 ± 0.8	↑			25 ± 0.9	↑	19 ± 1.3	↑					
Sr	0.026	802–1365	1247 ± 14	*	1365 ± 29	1048 ± 11	1002 ± 18	*	909 ± 13	*	804 ± 5	714 ± 8	862 ± 11	828 ± 19	912 ± 12
			1137 ± 19	↓			830 ± 3	↓	802 ± 40	↓					
Zn	0.17	14–107	61 ± 1.0	*	36 ± 2.7	107 ± 4.3	35 ± 1.3	*	34.0 ± 1.0	*	35.2 ± 1.1	14.6 ± 0.6	16.6 ± 0.8	14.4 ± 0.3	15.2 ± 0.4
			48 ± 1.2	↓			41 ± 1.6	↑	25.4 ± 2.1	↓					
Ash,	-	19–28	23.3 ± 0.2		20.9 ± 0.3	25.1 ± 2.1	20.2 ± 5.1		21.2 ± 2.5		28.2 ± 0.4	19.1 ± 0.9	20.1 ± 0.3	20.1 ± 1.0	20.3 ± 0.8
% DW			21.9 ± 0.3	↓			18.5 ± 0.5	↑	28.5 ± 0.3	↑					

LOQ, limit of quantification; ∆, concentration change; ↑, increase in concentration in the fertile reproductive phase compared to the sterile phase; ↓, decrease in concentration in the fertile reproductive phase compared to the sterile phase; St. 1–St. 9, the sampling stations (details in Section 3.2). * *p* < 0.05 based on a comparison between fertility and sterility.

**Table 2:** Pearson’s correlation matrix for the different trace metals in the *F. vesiculosus* samples from the Arctic region. Significant correlations (*p* < 0.5) are marked in bold.

Elements	Al	As	Ba	Ca	Co	Cu	Fe	Mg	Mn	Rb	Sr	Zn
Al	1											
As	−0.375	1										
Ba	−0.242	**−0.650**	1									
Ca	0.508	−0.222	−0.183	1								
Co	0.517	−0.348	0.074	0.315	1							
Cu	**0.789**	−0.349	−0.202	0.315	**0.538**	1						
Fe	**0.889**	−0.420	−0.114	0.431	0.482	**0.947**	1					
Mg	**0.793**	**−0.685**	0.204	0.282	0.338	**0.618**	**0.741**	1				
Mn	0.361	**−0.583**	**0.532**	0.151	0.495	0.043	0.197	**0.519**	1			
Rb	0.201	**−0.701**	0.394	0.216	0.110	−0.018	0.088	0.402	0.307	1		
Sr	**0.559**	−0.293	−0.200	**0.577**	**0.586**	0.383	0.380	0.270	0.424	0.066	1	
Zn	**0.775**	−0.073	−0.459	0.414	**0.578**	**0.922**	**0.877**	0.423	−0.038	−0.169	0.466	1

**Table 3:** Maximum concentration (mg/kg) and the daily dose (mg/day) of elements found in *F. vesiculosus* from different sampling sites, and a comparison with daily dose risk estimators for a 70 kg man and with the nutritional requirements.

Element	Sampling Site with a Maximum Concentration	Reproductive Phase	Maximum Concentration	Daily Dose for 3.3 g Consumption	Daily Dose for 12.5 g Consumption	Daily Dose from Risk Estimators	Daily Nutritional Requirements
Al	IS, St. 1	Fertile	724	2.39	9.03	10 ^1^	
As total	BS, St. 4	Sterile	58	0.19	0.72	0.15 ^1^ (inorganic)	
Ca	IS, St. 1	Sterile	30,093	99	375	2500 ^2^	1000 ^3^
Co	WS, St. 9	Fertile	2.0	0.007	0.025	30 ^5^	10 ^5^
Cu	IS, St. 2	Sterile	16.6	0.05	0.21	5 ^2,5^	0.9 ^4^/1.0 ^5^
Fe	IS, St. 2	Sterile	2217	7.3	27.7	45 ^5^	10 ^3,5^
Mg	IS, St. 1	Fertile	11,571	38	144	800 ^5^	400 ^5^
Mn	WS, St. 7	Fertile	156	0.52	1.95	11 ^5^	2.7 ^3^/2.0 ^5^
Zn	IS, St. 2	Sterile	107	0.35	1.33	25 ^2^	12 ^3,5^

^1^ PTWI, provisional tolerable weekly intake; ^2^ UL, tolerable upper intake level; ^3^ PRI, population reference intake; ^4^ AI, adequate intake; ^5^ [71].

**Table 4:** Characterization of the sampling stations.

Sea Area	Sampling Site	Coordinates	Reproductive Phase	Station (No. on the Map; Figure 7)	Mean Water Temperature, °C	Range of Salinity, ‰
Irminger Sea	Fossvogur Bay	64.120887 N 21.930663 W	Fertile	1	13.9	29.8 ± 0.3
Irminger Sea	Fossvogur Bay	64.120978 N 21.929122 W	Sterile	1a	4.0	34.9 ± 0.1
Irminger Sea	Fossvogur Bay	64.120887 N 21.930663 W	Sterile	1	4.0	35.2 ± 0.3
Irminger Sea	Seltjarnarnes Peninsula	64.15035 N 21.97255 W	Sterile	2	3.9	34.5 ± 0.6
Norwegian Sea	Sudspissen Cape	69.627168 N 18.912621 E	Fertile	3	9.1	33.1 ± 0.3
Norwegian Sea	Sudspissen Cape	69.627168 N 18.912621 E	Sterile	3	6.3	35.0 ± 0.2
Barents Sea	Teriberskaya Bay	69.184068 N 35.259487 E	Fertile	4	9.1	20.3 ± 0.4
Barents Sea	Teriberskaya Bay	69.184068 N 35.259487 E	Sterile	4	4.2	25.4 ± 0.6
Barents Sea	Teriberskaya Bay	69.173088 N 35.168468 E	Fertile	4	11.2	15.1 ± 0.4
Barents Sea	Zelenetskaya Bay	69.117150 N 36.070790 E	Fertile	5	10.3	31.5 ± 0.5
White Sea	Bolshoy Gorely island	66.31376 N 33.612736 E	Fertile	6	12.8	22.8 ± 0.7
White Sea	Matrenin Island	66.30945 N 33.631920 E	Fertile	7	14.1	15.3 ± 0.2
White Sea	Malyy Andronin Island	66.333374 N 33.766743 E	Fertile	8	13.0	25.9 ± 0.4
White Sea	Pezhostrov Island	66.273315 N 33.934406 E	Fertile	9	17.2	22.2 ± 0.1

## Data Availability

The data are available on request from the corresponding author.
